# Changes in Body Image and Pain in Patients with Cushing Syndrome: A Predictor for Depression and Vice Versa

**DOI:** 10.1210/jendso/bvaf226

**Published:** 2026-02-17

**Authors:** Franziska Bednarski, Leah T Braun, Frederick Vogel, Christian Adolf, Elisabeth Nowak, Stephanie Zopp, German Rubinstein, Felix Beuschlein, Petra Zimmermann, Martin Reincke, Heike Künzel

**Affiliations:** Department of Medicine IV, University Hospital, LMU Munich, Endocrinology, Munich 80336, Germany; Department of Medicine IV, University Hospital, LMU Munich, Endocrinology, Munich 80336, Germany; Department of Medicine IV, University Hospital, LMU Munich, Endocrinology, Munich 80336, Germany; Department of Medicine IV, University Hospital, LMU Munich, Endocrinology, Munich 80336, Germany; Department of Medicine IV, University Hospital, LMU Munich, Endocrinology, Munich 80336, Germany; Department of Medicine IV, University Hospital, LMU Munich, Endocrinology, Munich 80336, Germany; Department of Medicine IV, University Hospital, LMU Munich, Endocrinology, Munich 80336, Germany; Department of Medicine IV, University Hospital, LMU Munich, Endocrinology, Munich 80336, Germany; Department of Endocrinology, Diabetology and Clinical Nutrition, University of Zurich (UZH), 8091 Zurich, Switzerland; The LOOP Zurich - Medical Research Center, 8044 Zurich, Switzerland; Department for General, Visceral and Transplant Surgery, University Hospital, LMU Munich, Munich 80336, Germany; Department of Medicine IV, University Hospital, LMU Munich, Endocrinology, Munich 80336, Germany; Department of Medicine IV, University Hospital, LMU Munich, Psychosomatic, Munich 80336, Germany

**Keywords:** Cushing syndrome, depression, body image perception, pain

## Abstract

**Context:**

Cushing syndrome (CS) is a rare disease with severe physical and psychological effects, especially depression, which often persists despite biochemical remission.

**Objective:**

The aim of this study was to identify possible prognostic markers for depression in patients with CS.

**Methods:**

Biochemical and clinical data from the German Cushing registry were retrospectively analyzed, along with the Beck Depression Inventory II and Patient Health Questionnaire-9 depression scores. Pain and body image were collected using the Fragebogen zum eigenen Körpers (Questionnaire on one’s own body) (FBeK), Fragebogen Körperbild (Questionnaire on body image) (FKB-20), and a numerical analogue scale for pain. The study included 90 patients with CS and 200 controls with clinically suspected but biochemically excluded CS. For detailed analysis, 15 CS patients with and 10 CS patients without depression were examined.

**Results:**

Manifest depression was diagnosed in 44% of CS patients. There were no significant differences in anthropometric or biochemical parameters between patients with and without depression. Patients with persistent depression exhibited significantly more negative body image results (FBeK: scales 1 and 2, *P* < .001; FKB-20: vital body dynamics, *P* < .001; and negative body perception, *P* = .02). They also reported significantly higher pain levels (*P* < .001). A significant correlation between pain and body image disturbances was found in most questionnaires. In comparison to depressive and obese groups from the literature, CS patients present with a complex body image disturbance.

**Conclusion:**

Pain and body image are relevant factors influencing depressive mood. CS treatment should address the physical changes and the body's lack of energy, strength, and ability to cope with everyday life.

Cushing syndrome (CS) is a rare but potentially life-threatening endocrine disorder characterized by endogenous hypercortisolism. CS is classified as adrenocorticotropin (ACTH) dependent or independent and further categorized by origin into pituitary, adrenal, or ectopic forms. Patients suffer from various clinical signs and comorbidities. Besides somatic comorbidities such as hypertension or diabetes, depression and other psychiatric disorders commonly occur in this group of patients [1]. Patients with CS have been described as being affected by mental disorders more than patients with other endocrine diseases [2]. Current reviews underline depression as one of the most common symptoms in 60% to 80% of patients with CS—even as an early symptom [3, 4]. Up to 80% fulfill the criteria of an anxiety disorder. Other symptoms or disorders such as mania, cognitive impairment, sleep disturbances, and loss of libido were described [5]. Additionally, in a long-term follow-up over 5 years, 372 patients with Cushing disease (CD) were found to need more antidepressive and anxiolytic medication as well as analgesics compared to healthy controls [6]. Santos et al [4] in 2019 reported an impaired quality of life (QoL) in these patients, mainly in women. The authors found depression, anxiety, and poor coping strategies to be worsening factors for QoL. Socioeconomic burden in these patients is described to be high. Patients’ relationships are negatively influenced by the disease and they are at risk to be unemployed or retired [7]. Risk factors are female sex, low educational status, and comorbidities. Despite the high prevalence and the known impairment of QoL, there is still little knowledge about the exact causes of depression in CS. Conversely, depression alters physical perceptions. Studies have shown a reciprocal relationship between depression and pain as well as between dissatisfaction with one's own body due to obesity and depression [8, 9]. Disfiguring physical changes—like round face, dorsocervical fat pad, striae lividae, obesity—and pain are very frequent in patients with CS [10, 11]. However, only a few studies to date have focused on body image or pain in patients with CS. A significantly more negative body image as well as a positive correlation between body image disturbance and depression in patients with CD has been demonstrated [12, 13]. A relevant feature of patients’ self-perception is that physical fitness and the ability to carry out everyday activities is particularly impaired [13]. There was also low body satisfaction and self-esteem in patients with CS in remission [14]. We hypothesized that body image and pain are key factors in the emergence of depression in patients with CS. Our aim was to evaluate the body image of patients with persistent depression in CS in comparison to patients with CS without depression. Understanding the body image of the individual patient could offer a treatment approach in therapy and thus improve mental and physical well-being.

## Materials and Methods

All patients and controls included in this monocentric longitudinal cohort study were part of the German Cushing registry (CUSTODES) and were recruited at the LMU Hospital in Munich. Details regarding the registry have been previously described [15-17]. Written informed consent was obtained by all participants. The study protocol was approved by the ethics committee and complied with the Declaration of Helsinki.

### Study Group

**Figure 1.**
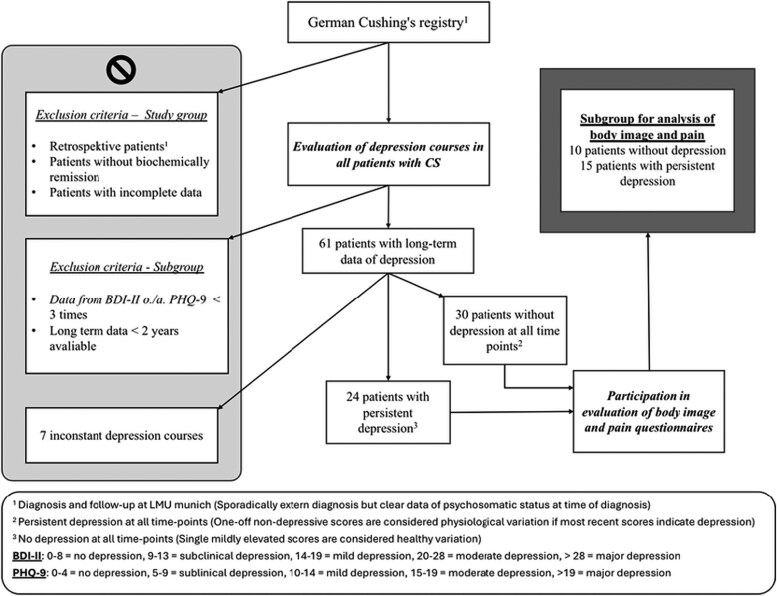
Workflow for patient selection.

The study included all patients enrolled in the Cushing registry at LMU from its foundation in 2012 until July 2022. An overview of the workflow for patient selection is shown in Fig 1. Patients without biochemical remission after surgery as well as those with incomplete data—especially missing depression scores—were excluded. The diagnosis of CS was established as described in the guidelines [18]. All patients underwent standardized procedures for examination. Alongside clinical and biochemical evaluations, participants were asked to complete questionnaires assessing psychological stress. Evaluations were conducted at the time of diagnosis, 6 months after treatment, and subsequently during annual follow-ups. In most cases, data were available for at least 2 time points.

Data from the Cushing registry are available for 90 patients with confirmed CS. The control group consists of 200 patients, in whom CS could be ruled out. However, this does not refer to a healthy cohort because patients presented due to suspected CS.

In a second step we investigated the long-term depression courses of all registry patients with CS to identify individuals with distinct depression courses for targeted considerations (see Fig 1). Patients were included if data were available at least at 3 time points over a period of 2 or more years. For this analysis, only the 24 patients with persistent depression and 30 patients without depression at all assessed time points were considered. In this subsample, additional questionnaires were administered. The response rate was only about 50% and, in a few cases, the newest Beck Depression Inventory II (BDI-II) did not fit in the previously shown progression. At least data from 15 patients with persistent depression and 10 patients without depression in the long-term were available.

To enable a more nuanced analysis of body image, data from various cohorts—reported in related publications or manuals of these questionnaires—were included. For the “Questionnaire on one's own body” (FBeK), reference data from a healthy cohort were used. In the evaluation of the “Questionnaire on body image” (FKB), data from a healthy cohort, a depressive cohort without CS, and an overweight cohort were considered.

### Questionnaires on Depression, Anxiety, Pain, and Body Image

#### Depression and anxiety

Depression was assessed using both the short form of the Patient Health Questionnaire (PHQ-9) and the BDI-II, which are self-reporting questionnaires designed to evaluate the presence and severity of depressive disorders [19, 20]. Anxiety was assessed using the Generalized Anxiety Disorder-7 (GAD-7), a validated screening and clinical assessment tool for generalized anxiety disorder [21]. Standard threshold values were applied for all questionnaires.

#### Pain

Pain intensity was assessed using the numerical analogue scale (NAS), corresponding to item 11 of the “German pain questionnaire” [22]. Patients rated their pain on a scale from 0 (no pain) to 10 (worst imaginable pain). The assessment included 4 aspects: current pain intensity, average pain as well as maximum pain intensity over the past 4 weeks, maximum pain intensity, and the level of pain considered tolerable in the long term.

#### Body image

The FKB-20 was used to assess body image disturbances and subjective body perception [23]. This questionnaire consists of 20 items each rated on a 5-point Likert scale ranging from “does not apply at all” to “applies completely.” Two different scales can be derived from 10 items each: “negative body perception” (NBP) and “vital body dynamics” (VBD). Higher scores on the NBP scale indicate a pronounced negative attitude toward one's body, whereas higher scores on the VBD scale reflect “perceived physical health, strength, and activity.” The FBeK was used to assess “differential aspects of body experience” [24]. Patients responded to 52 items related to body perception using a dichotomously scale of “agree” and “disagree.” Data were analyzed using a validated 3-scale model, selected due to better data availability and to enable comparisons with reference data from various clinical and nonclinical populations. This questionnaire focuses on the following 3 aspects: Scale 1 shows “insecurity and discomfort”; scale 2, “attractiveness and self-confidence”; and scale 3, “Accentuation of the body/sensitivity.”

### Statistical Methods

All collected data were analyzed descriptively and in group comparisons. To evaluate the questionnaires, patients were categorized into various stages of severity—of depression and anxiety disorder—based on validated literature references specific for each questionnaire. Due to the lack of normal distribution, nonparametric tests were used. Metric data are presented using percentiles, and frequencies are shown in percentage.

The Mann-Whitney *U* test was used to determine significant rank sum differences between patients with CS and the control group, as well as between patients with and without depression. For testing rank sum differences between the 3 subgroups of CS, the Kruskal-Wallis test was used. Spearman rho (ρ) was employed to assess correlations between various parameters. Statistical analyses were conducted using IBM SPSS software, with statistical significance defined as a *P* value less than .05. No adjustments were made for multiple testing or potential confounding.

## Results

### Data from Cushing´s Registry at Time of Diagnosis

Data from 90 patients with CS (61 CD, 8 ectopic CS, 21 adrenal CS) and 200 controls were available. Sociodemographic and biochemically data are shown in Table 1. It took on average 2 years from first symptoms to final diagnosis of CS.

**Table 1.** Sociodemographic and basic biochemical results

	CS	Exclusion of CS	*P*
**N**	**90**	**200**	
**Subtype**	68% pituitary 9% ectopic 23% adrenal	—	—
**Sex,** women	73%	71%	.825
**Age at diagnosis, y**	51 (37-58)	35 (24-48)	**<**.**001**
**Time to diagnosis, y**	2 (1-5)	—	—
**HbA_1c_, %**	5.8 (5.4-6.4)	5.3 (5.1-5.7)	**<.001**
**Cholesterol,** mg/dL	214 (185-244)	199 (175-223)	**.011**
**Triglycerides,** mg/dL	122 (83-201)	120 (82-182)	.481
**HDL,** mg/dL	61 (50-70)	54 (42-67)	**.012**
**LDL,** mg/dL	120 (101-160)	120 (98-142)	.114
**UFC,** µg/24 h	551 (249-839)	147 (87-231)	**<.001**
**Serum cortisol,** µg/dL	20.5 (15.5-28.6)	8.9 (6.7-13.1)	**<.001**
**LNSC,** ng/mL	9.2 (4.6-16)	1.1 (0.7-1.9)	**<.001**
**LDDST,** µg/dL	14.4 (7.4-24.4)	1.0 (0.8-1.3)	**<.001**
**BMI**	29 (25-33)	32 (27-39)	**.023**
**Hypertension**	87%	57%	**<.001**
**Type 2 diabetes**	31%	15%	**.002**
**Dyslipidemia**	69%	52%	**.013**
**Osteoporosis**	25%	4%	**<.001**

Shown are median and interquartile ranges; bold numbers indicate statistical significance (*P* < .05).

Abbreviations: BMI, body mass index; CS, Cushing syndrome; HbA_1c_, glycated hemoglobin A_1c_; HDL, high-density lipoprotein; LDL, low-density lipoprotein; LNSC, late-night salivary cortisol; LDDST, 1-mg low-dose dexamethasone suppression test; UFC, urinary free cortisol.

#### Prevalence of depression

A total of 44% of patients with CS—measured by BDI-II—suffered from depression at time of diagnosis (Table 2). There was a clear discrepancy in the prevalence of depression as reported by patients. Only 30% of patients with CS reported a history of depression.

**Table 2.** Prevalence of depression and anxiety at time of diagnosis

	CS	Exclusion of CS	*P*
**N**	**90**	**200**	
**Depression self-reported** *^a^*	30%	33%	.656
**Depression diagnostic** *^b^*	44%	60%	**.02**
**Anxiety disorders**	6%	4%	.349
**BDI-II score**	11 (7-24)	17 (8-28)	.323
**GAD-7 score**	6 (3-12)	8 (4-13)	.304
**PHQ-9 score**	8 (4-14)	11 (5-15)	.243

Shown are median and interquartile ranges; bold numbers indicate statistical significance (*P* < .05).

Abbreviations: BDI-II, Beck Depression Inventory II; CS, Cushing syndrome; GAD-7, Generalized Anxiety Disorder-7, PHQ-9, Patient Health Questionnaire-9.

^a^Anamnestic.

^b^Diagnostic BDI-II score greater than 13.

#### Comparison of patients with Cushing syndrome and controls

Patients with CS were significantly older at time of diagnosis than patients in the group without CS (age 50.5 vs 35; *P* < 0001). A total of 73% of patients with CS were female, in the control group the proportion of female participants was 71%. At time of diagnosis, patients with CS suffered significantly more often from hypertension, diabetes, or osteoporosis in comparison to patients with exclusion of CS (see Table 1). However, obesity and depression were more frequent in controls compared to patients with CS.

#### Comparison of adrenal, ectopic, and pituitary Cushing syndrome

The comparison between the subtypes of CS revealed no statistically significant differences in biochemical parameters (Table 3). Patients with CS showed significant biochemical differences from controls, including elevated cortisol levels (urinary free cortisol [UFC] and serum cortisol), pathological late-night salivary cortisol and low-dose dexamethasone suppression test results, as well as increased ACTH levels (all *P* < .001). No differences were observed between CS subgroups in terms of depression severity, body mass index (BMI), or waist circumference, except for a statistically significant difference in hip circumference (*P* < .001). Sex distribution analysis revealed no statistically significant differences (*P* = .97), aligning approximately with that of the overall cohort across the subgroups. When analyzing patients with CS and controls separately by depression status at time of diagnosis, no statistically significant differences were found in biochemical parameters, sex distribution, or body measurements (data not shown).

**Table 3.** Baseline characteristics in group comparison: pituitary vs ectopic vs adrenal

	CD	Ectopic CS	Adrenal CS	Controls	*P*
**N**	**61**	**8**	**21**	**200**	
**BMI**	30 (25-33)	27 (24-28)	29 (26-36)	32 (27-39)	.**23**
**Waist circumference,** cm	103 (92-112)	98 (83-107)	106 (89-112)	101 (87-117)	.699
**Hip size,** cm	104 (97-113)	99 (96-114)	111 (101-117)	114 (104-125)	**<.001**
**Sex,** female	74%	75%	71%	71%	.968
**UFC,** µg/24 h	483 (286-827)	2014 (706-4118)	303 (144-620)	147 (87-231)	**<.001**
**Serum cortisol,** µg/dL	20.7 (15.8-28.6)	45.0 (27.0-67.3)	13.3 (7.8-20.0)	8.9 (16.7-13.1)	**<.001**
**LNSC,** ng/mL	9.2 (4.5-16.2)	22.1 (13.7-117.7)	7.6 (3.8-13.2)	1.1 (0.7-1.9)	**<.001**
**LDDST,** µg/dL	13.4 (6.3-20.4)	53.4 (39.2-58.0)	14.5 (7.4-20.9)	1.0 (0.8-1.3)	**<.001**
**ACTH,** pg/mL	60.5 (36.8-84.0)	134 (49.8-353.0)	2.0 (2.0-4.0)	12.0 (8.0-18.0)	**<.001**
**BDI-II score**	11 (7-23)	10 (7-25)	16 (5-28)	17 (8-28)	.058
**PHQ-9 score**	8 (4-13)	10 (6-20)	12 (3-15)	11 (5-15)	.243

Shown are median and interquartile ranges; bold numbers indicate statistical significance (*P* < .05).

Abbreviations: ACTH, adrenocorticotropin; BDI-II, Beck Depression Inventory II; CD, Cushing disease; CS, Cushing syndrome; LDDST, 1-mg low-dose dexamethasone suppression test; LNSC, late-night salivary cortisol; PHQ-9, Patient Health-Questionnaire 9; UFC, urinary free cortisol.

### Patients with Long-term Follow-up: Analysis of Body Image and Pain

Long-term data on depression was extracted for 61 patients from the Cushing registry. Final data, including current BDI-II scores as well as measures of body image and pain, were available for 15 patients with persistent depression and 10 patients without depression.

Patients were on average observed for 4 years (interquartile range, 3-5 years).

#### Baseline characteristics of patients with and without depression

Sociodemographic, clinical, and biochemical aspects at time of diagnosis did not differ between patients with and without depression, especially not in BMI, sex, and cortisol concentrations (Table 4). Scores for the BDI-II, GAD-7, and PHQ-9 were significantly higher in patients with persistent depression at time of diagnosis and follow-up (*P* < .001) (Table 5).

**Table 4.** Sociodemographic and basic biochemical results in the subgroup at time of diagnosis

	CS without depression	CS with persistent depression	*P*
**N**	**10**	**15**	
**Subtype**	90% pituitary 10% ectopic	53% pituitary 47% adrenal	—
**Sex,** female	90%	87%	.9
**Age at diagnosis,** y	49 (33-60)	48 (39-55)	.7
**Time to diagnosis,** y	1 (1-7)	3 (1-7.5)	.5
**HbA_1c_,** %	5.8 (5.3-7.1)	5.9 (5.4-6.7)	.8
**Cholesterol,** mg/dL	226 (169-264)	194 (174-250)	.8
**Triglycerides,** mg/dL	115 (72-183)	133 (79-201)	.7
**HDL,** mg/dL	69 (55-83)	58 (50-67)	.2
**LDL,** mg/dL	139 (82-162)	116 (97-147)	.5
**BMI**	32 (28-41)	28 (26-34)	.2
**Hypertension**	89%	86%	.9
**Diabetes mellitus**	40%	29%	.7
**Dyslipidemia**	67%	64%	.9
**Osteoporosis**	22%	39%	.6
**UFC,** µg/24 h	410 (175-685)	324 (268-766)	.9
**Serum cortisol,** µg/dL	20 (15-30)	18 (11-27)	.6
**LNSC,** ng/mL	6 (3-14)	9 (3-26)	.7
**LDDST,** µg/dL	11 (7-23)	15 (9-18)	≥.999

Shown are median and interquartile ranges; Bold numbers indicate statistical significance (*P* < .05).

Abbreviations: BMI, body mass index; CS, Cushing syndrome; HbA_1c_, glycated hemoglobin A_1c_; HDL, high-density lipoprotein; LDL, low-density lipoprotein; LDDST, 1-mg low-dose dexamethasone suppression test; LNSC, late-night salivary cortisol; UFC, urinary free cortisol.

**Table 5.** Scores of depression and anxiety questionnaires in the subgroup at time of diagnosis

	CS without depression	CS with persistent depression	*P*
**N**	**10**	**15**	
**Anxiety disorders**	0%	14%	.6
**BDI II score**	7.5 (3-9.5)	26 (20-44)	**<**.**001**
**GAD-7 score**	2 (1-5)	11 (5-17)	**<.001**
**PHQ-9 score**	5 (3-6)	13 (12-16)	**<.001**

Shown are median and interquartile ranges; bold numbers indicate statistical significance (*P* < .05).

Abbreviations: BDI-II, Beck Depression Inventory II, CS, Cushing syndrome; GAD-7, Generalized Anxiety Disorder-7; PHQ-9, Patient Health-Questionnaire 9.

#### Pain in patients with and without depression

The evaluation of an NAS of pain showed statistically significant higher values in all aspects for the depressive cohort (Table 6). This indicated greater pain at the time of the survey, as well as on average in the last 4 weeks. The average pain was 0 out of 10 (0-1) in patients without depression and 5 out of 10 [3-7] in patients with depression. The maximum pain intensity during this period was also higher in the depressive group (0 vs 7; *P* < .001). Patients with persistent depression also reported a higher maximum tolerable pain intensity.

**Table 6.** Body image and pain on patients with and without long-term depression

	CS without depression	CS with persistent depression	*P*
**N**	**10**	**15**	
**BDI-II score**	5 (1.5-6)	25 (22-37)	**<**.**001**
**FBeK: scale 1**	4.5 (3-6)	11 (9-15)	**<.001**
**FBeK: scale 2**	4.5 (2-11)	2 (1-4)	**.04**
**FBeK: scale 3**	9.5 (6-12)	12 (10-14)	.06
**FKB-20: VBD**	31 (27-35)	19 (14-24)	**<.001**
**FKB-20: NBP**	24 (12-29)	36 (22-43)	**.02**
**NAS 1**	0.5 (0-1)	4 (2-6)	**<.001**
**NAS 2**	0 (0-1)	5 (3-7)	**<.001**
**NAS 3**	0 (0-1)	7 (5-9)	**<.001**
**NAS 4**	2 (1-2)	3 (2-4)	**.03**

Shown are median and interquartile ranges; bold numbers indicate statistical significance (*P* < .05).

Abbreviations: CS, Cushing syndrome; FBeK, Fragebogen zum eigenen Körpers (Questionnaire on one’s own body); FKB-20, Fragebogen Körperbild (Questionnaire on body image); NAS, numeral analogue scale; NAS 1, current pain intensity, NAS 2, average pain intensity in the past 2 weeks, NAS 3, highest pain intensity in the past 4 weeks, NAS 4, pain intensity that would be tolerable in the long term; NBP, negative body perception; scale 1, insecurity and discomfort, scale 2, attractiveness and self-confidence, scale 3, accentuation of body/sensitivity; VBD, vital body dynamics.

#### Body image in patients with and without depression

Patients with depression had significantly higher scores on the FBeK scale 1 (4.5 vs 11; *P* < .001) (see Table 6). These findings indicate “insecurity and discomfort,” which reflects a lack of sensitivity and the desire for physical experience [24]. Furthermore, they suggest a disturbance in body image perception and a rejection of both the body's appearance and its physiological responses. Patients also had lower scores on scale 2, which indicates a lower feeling of “attractiveness and self-confidence” in this group (4.5 vs 2; *P* < .001). There were no statistically significant differences between the groups on scale 3 “accentuation of the body/sensitivity.” Similar findings applied for scales of the FKB-20 (see Table 6). The group with persistent depression achieved significantly lower scores on the VBD scale (19 vs 31; *P* < .001). The NBP was significantly more pronounced (24 vs 36; *P* = .020). Age and sex did not correlate with results of the body image scores as well as perception of pain. However, there was a distinct association between pain and body image. A statistically significant negative correlation was found between pain and VBD (rs = −0.647; *P* < .001). In contrast to this, a positive correlation for pain and NBP (rs = 0.441; *P* = .005).

#### Comparison of body image questionnaires with data from the literature

**Figure 2.**
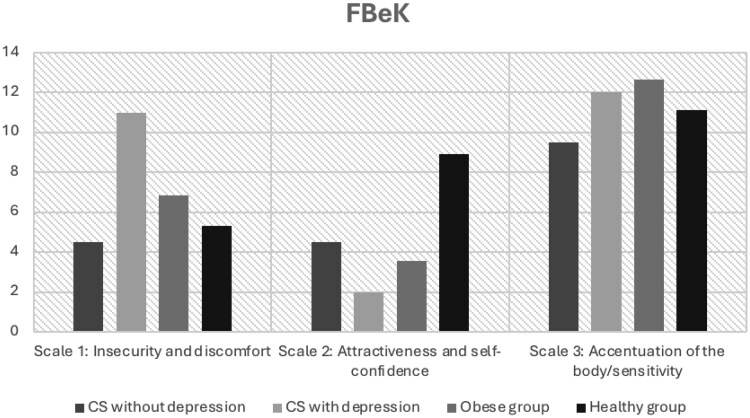
Comparison of “Questionnaire on one’s own body” (FBeK) values of Cushing syndrome (CS) patients with healthy and obese groups [24].

For the analysis of the FBeK, we contrasted the scores of our patients with CS—with persistent depression and without depression—with literary results of an obese and of a healthy group (Fig. 2). On scale 1—“insecurity and discomfort”—patients with persistent depression had higher scores than the obese group. In contrast, patients with CS without depression exhibited scores within the range of the healthy control group. On scale 2—“attractiveness and self-confidence”—the scores of patients with persistent depression were similar to those of the obese cohort, whereas the nondepressed CS group scored below the healthy control group from the literature. Minimal differences were observed between the groups on scale 3—“Accentuation of the body/sensitivity.”

**Figure 3.**
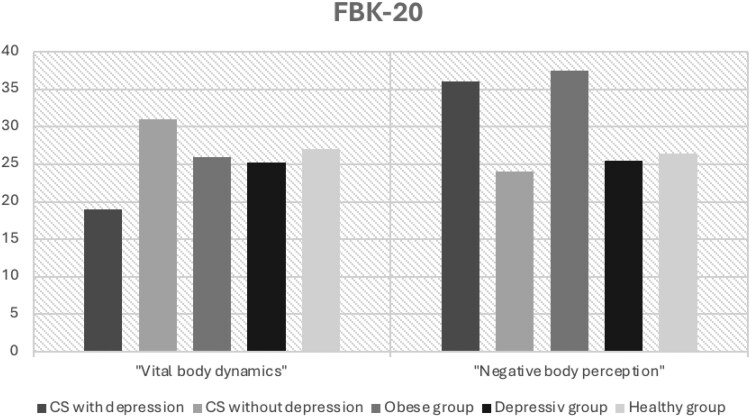
Comparison of “Questionnaire on body image” (FKB-20) values of Cushing syndrome (CS) patients with healthy and obese cohorts [23].

The FKB-20 enables comparisons with a group of patients with depression but without CS as well as an obese and a healthy group (Fig. 3). In the VBD domain, scores of patients with CS and persistent depression were significantly lower than those of both the purely depressive and obese groups. Patients with CS without depression also scored lower than the healthy group, aligning more closely with the values of the obese and purely depressive groups. On the scale measuring NBP, patients with CS and persistent depression had scores similar to the obese group and higher than those of the purely depressive group. Scores for patients with CS without depression were approximately equivalent to those of the healthy group.

## Discussion

Depression is a well-known comorbidity of CS. The discrepancy in depression prevalence between self-reported and diagnostic assessment highlights the importance of depression screening in patients with CS. In many studies a persistence of depressive symptoms has been reported—even after biochemical remission—but the reason remains unclear [25, 26].

This study clearly demonstrates that, apart from the cortisol levels defining the disease, biochemical parameters did not differ between patients with CS and controls. No statistically significant differences were observed between CS subgroups either. However, the study identified a significant influence of negative body image and pain on the persistence of depressive symptoms.

Most studies see the dysregulated hypothalamic-pituitary-adrenal axis as a main pathophysiological factor for the depressive symptoms [27, 28]. Comparison of ACTH-dependent and ACTH-independent CS did not reveal any statistically significant difference in the degree of depressive symptoms. Commonly the high concentration of cortisol is considered to be the main factor causing depression in CS. There is also disagreement in the literature regarding the correlation between depressive symptoms and elevated cortisol concentrations in CS. While Sonino et al demonstrated a correlation between depression and elevated UFC concentrations in patients with CS, the study by Cohen—as well as this study—did not find a correlation between the severity of depression and peripheral cortisol concentrations [29, 30]. The initial part of the analysis aimed to explore general potential determinants associated with the presence of depression. Furthermore, the influence of the subtype was assessed.

We did not find a statistically significant difference between controls and patients with CS nor between the subgroups of CS in comorbidities, biochemical, or anthropometrical parameters. This might suggest that the dysregulation of the hypothalamic-pituitary-adrenal axis is not the only factor for the development of depressive symptoms. Additionally, the prevalence of depression was markedly higher in patients with CS and controls—44% and 60%—compared to 19% in the general population [31]. These findings suggest the possibility that the cause of depression may be related to a factor common both to patients with CS and controls. One potential factor is obesity, as BMI did not differ significantly between the groups and was clearly within the obese range in both.

The topic of body image was initially mentioned in 2013 by Alcalar et al [12], who investigated 40 patients with CD after transsphenoidal surgery with and without remission, as well as 40 healthy controls. The authors’ findings revealed a significantly more negative body image in patients with CD, regardless of remission status. Similarly, Sharma et al [13] in 2018 reported body image disturbances in 50% of CD patients and low self-esteem in 60% of patients, with a notable prevalence of a desire for cosmetic camouflage. In a study involving 63 patients with CS in remission, Vermalle et al [14] in 2018 came to similar conclusions regarding body image concerns. It is important to note that in the present study no differences in BMI or other anthropometric measurements were observed between patients with CS and controls—exclusion of CS—neither at the onset of the illness nor during follow-up assessments in remission. This suggests that dissatisfaction with body image is not solely attributable to external physical factors. For comparison, literature data of individuals with depression and obesity were analyzed. Patients with persistent depression exhibited the highest scores on scale 1 of the FBeK among all literature comparison groups—including an obese group [24]. This finding highlights an increased level of “insecurity and discomfort” in these patients. One possible explanation for this phenomenon are the physical changes associated with CS, which may exacerbate dissatisfaction with one's own body and amplify psychological distress. Notably, nondepressed patients with CS achieved scores on scale 1 comparable to those of a healthy cohort. This observation raises an interesting question: Do these individuals possess resilience factors that protect them from body image–related distress? Further research is needed to better understand this ability and the underlying factors. Psychological resilience, coping skills, or social support might provide an explanation. These factors might also be useful for therapy. In contrast, overweight appears to have a certain influence on the perception of “attractiveness and self-confidence” (scale 2). In this domain, the scores of the nondepressed cohort with CS resembled those of an obese group. While scores of patients with CS with persistent depression are lower, they remain in a similar range. Meanwhile, a healthy control group achieved scores twice as high. Although body weight seems to exert an apparent influence, other factors may also play a decisive role. Notably, the 2 cohorts with CS differ significantly in this regard despite showing no differences in BMI. Analyzing the individual questions of this scale, in addition to weight-related concerns, factors such as perceived attractiveness, body satisfaction, pride, and self-care may also contribute [24]. Comparing the FKB-20 with literature values provides insight into the differences between CS-related depression and primary depression [23]. Patients with CS and depression exhibit the lowest scores in the VBD domain compared to all other groups. This suggests that disease-related factors associated with CS may play a role. Analyzing individual items on this scale highlights potential influencing factors, including perceived physical robustness, overall health, energy levels, and sexual satisfaction. Furthermore, patients with CS without depression achieve the highest scores on this scale across all groups, further supporting the hypothesis of resilience factors in body perception. In the domain of NBP, overweight appears to exert a central influence. Patients with persistent depression exhibit values similar to the obese cohort. However, it remains unclear why the overweight patients with CS without depression are in the range of the healthy and the depressed group. Again, an examination of the individual questions provides clues. The main influences in this area are physical defects, feeling good in one's own body, disgust with oneself, and the desire for a different body.

Pain was reported by 83% of patients, making it one of the most common symptoms in individuals with CS [32]. Moreover, studies have shown that pain often persists even after successful treatment [33]. Long-term studies have reported a significantly reduced QoL in patients with CS, with pain being a major contributing factor [26]. A close bidirectional relationship between pain and depression is described [8]. Both conditions are mediated by similar neurotransmitters within shared brain structures [34]. Noradrenaline for example has an inhibitory effect on pain within the amygdala and is one of the transmitters targeted by antidepressive treatment [35]. To our knowledge no studies to date have specifically investigated the severity and intensity of pain and its psychological effect in the context of CS. In our analysis we found a statistically significant difference in pain scores between patients with and without depressive symptoms in the long term. Furthermore, a significant correlation was observed between pain and body image questionnaires. These findings suggest that pain may be a substantial contributing factor to body image disturbance and persistent depression. None of the other investigated factors like weight, comorbidities such as diabetes, or endocrine parameters were found to correlate with persistent depression. Therefore, we strongly recommend screening patients even after biochemical normalization of CS for pain as well as for depression. These conditions have a mutual worsening influence and impair patients’ daily life.

A distinguishing feature of our research is the well-characterized patient cohort representing all forms of CS, which has been longitudinally studied according to standardized protocols. The use of different questionnaires assessing body image, as well as the analysis of pain across 4 categories of the visual analog scale, provides a more differentiated perspective on the topics of body image and pain. Limitations of this study are especially the monocentric patient population as well as the small number of patients for the second part of the analysis. In this context, the uneven distribution of subgroups resulting from the small sample size may have introduced bias in the comparison between patients with and without depression.

Further limitations regarding the patient and control groups may include the fact that the control group does not represent a healthy population but rather an obese cohort, which may limit the comparability of the findings. Moreover, patients with CS were significantly older than controls, which may have affected the findings. However, no differences in depression status were observed.

Further studies in this area are necessary to obtain a more accurate understanding. In particular, the consistent collection of long-term data on depression, body image, and pain could contribute to better data availability. Therefore, this study might help in obtaining better insight into potential influencing factors of persisting depression after remission of active disease.

In conclusion, numerous factors contribute to the development of persistent depression in patients with CS. Biochemical factors, such as cortisol concentrations at the time of diagnosis, seem to play a marginal role in our population. Obesity, as measured by BMI and body proportions, did not differ between patients with and without depression. However, the findings of our study indicate a statistically significant relationship between pain, body image perception disturbance, and the development of depressive symptoms. Additionally, comparisons of body image questionnaires with literature cohorts suggest the presence of potential protective factors that could play a central role in early psychosomatic therapy. Special emphasis should be placed on functionality in daily life and physical energy. Pain and dissatisfaction with physical changes, particularly with the altered functionality of the body in daily activities, should be promptly addressed. This approach may help to prevent the manifestation of persistent depression and its associated consequences.

## Data Availability

All datasets generated during and analyzed during the current study are not publicly available but are available from the corresponding author on reasonable request.

## Abbreviations

ACTH: adrenocorticotropin
BDI-II: Beck Depression Inventory II
BMI: body mass index
CD: Cushing disease
CS: Cushing syndrome
FBeK: Fragebogen zum eigenen Körper (Questionnaire on one’s own body)
FKB-20: Fragebogen Körperbild (Questionnaire on body image)
GAD-7: Generalized Anxiety Disorder-7
LDDST: 1-mg low-dose dexamethasone suppression test
LNSC: late-night salivary cortisol
NAS: numeral analogue scale
NBP: negative body perception
PHQ-9: Patient Health Questionnaire-9
QoL: quality of life
UFC: urinary free cortisol
VBD: vital body dynamics
